# A Comparison between Gadofosveset Trisodium and Gadobenate Dimeglumine for Steady State MRA of the Thoracic Vasculature

**DOI:** 10.1155/2014/625614

**Published:** 2014-06-29

**Authors:** G. Paul Camren, Gregory J. Wilson, Vikram R. Bamra, Khahn Q. Nguyen, Daniel S. Hippe, Jeffrey H. Maki

**Affiliations:** ^1^Radiology, University of Washington, Seattle, WA 98195, USA; ^2^Aurora Medical Group, Milwaukee, WI 53045, USA

## Abstract

*Purpose*. Retrospective comparison between gadofosveset trisodium and gadobenate dimeglumine steady state magnetic resonance angiography (SS-MRA) of the thoracic vasculature at 1.5T using signal-to-noise ratio (SNR) and vessel edge sharpness (ES) as markers of image quality. *Materials and Methods*. IRB approval was obtained. Twenty separate patients each underwent SS-MRA using high-resolution 3D ECG-triggered coronal IR-TFE at 1.5T approximately 3-4 minutes following 10 or 15 mL gadofosveset or 20 mL gadobenate. ROIs were placed in the right atrium, left ventricle, left atrium, ascending aorta, descending aorta, and right pulmonary artery to estimate SNR. Vessel ES was estimated as 20–80% rise distances from line intensity profiles in the left pulmonary vein, ascending aorta, and descending aorta. Data were analyzed using nonpaired Student's *t*-test (threshold for significance set at *P* < 0.05). *Results*. There was no significant difference in mean SNR for the gadofosveset or gadobenate groups (*P* values: 0.14 to 0.85). There was no significant difference in mean vessel ES for gadofosveset and gadobenate groups (*P* values: 0.17 to 0.78). *Conclusion*. High quality thoracic SS-MRA can be achieved with gadobenate dimeglumine, similar to that achieved with the blood pool agent gadofosveset trisodium provided that imaging is initiated quickly (3-4 min) after contrast injection.

## 1. Introduction

Contrast-enhanced magnetic resonance angiography (CE-MRA) is a commonly employed noninvasive technique for imaging the thoracic vasculature, as well as that of the neck, abdomen, and extremities. In the majority of cases, CE-MRA is performed as breath-hold, non-ECG-triggered “first pass” imaging (FP-MRA) after injection of an extracellular fluid (ECF) gadolinium based contrast agent (GBCA). The recently introduced “blood pool” GBCA gadofosveset trisodium (Ablavar; Lantheus Medical Imaging, Billerica, MA, USA) has found widespread applications in steady state MRA (SS-MRA), where its prolonged intravascular half-life and high T1 relaxivity allow for much longer acquisitions and subsequently much greater spatial resolution than what can be obtained with first pass CE-MRA [1–7]. In addition, the ability to image in the steady state makes the use of free-breathing, ECG-triggered SS-MRA in the thorax possible [8–10]. Such sequences have been shown to be particularly useful for thoracic MRA, where cardiac and respiratory motion otherwise cause significant blurring of the heart and aorta.

We perform a large volume of cardiothoracic and vascular MR examinations at our institution, and as part of this routine we perform FP- and SS-MRA, most often for evaluation and follow-up of ascending aortic pathology. For this we use either the blood pool agent gadofosveset trisodium, or the high relaxivity agent gadobenate dimeglumine (Multihance; Bracco Diagnostics, Princeton, NJ), with our choice of agent dictated in part by whether additional cardiac delayed enhancement (scar) imaging was desired as part of the study (in which case gadobenate dimeglumine was used) [11, 12]. Prior to gaining experience with thoracic SS-MRA, our expectation was that SS images using an extracellular agent would be of acceptable quality, but inferior to that of a blood pool agent. Our anecdotal experience, however, has noted surprisingly good quality of gadobenate dimeglumine SS-MRA, to the point that we now use the agents interchangeably unless there is a compelling reason to use one versus the other (e.g., cardiac delayed enhancement (gadobenate dimeglumine) or desire to image multiple territories (gadofosveset trisodium)). Thus this study was conceived to formally assess the difference (if any) in thoracic SS-MRA image quality between these two agents through the evaluation of intravascular signal-to-noise ratio (SNR) and vessel edge sharpness (ES).

## 2. Materials and Methods

With institutional review board approval, this parallel group study retrospectively examined 40 consecutive patients who underwent thoracic MRA that included SS-MRA (*n* = 20 gadofosveset trisodium; *n* = 20 gadobenate dimeglumine). Inversion recovery turbo field echo (IR-TFE) coronal 3D MRA exams with cardiac triggering and respiratory navigator gating were performed on a 1.5T Achieva (Philips Healthcare, Best, NL) scanner according to [8] with the following approximate parameters: FOV 360 × 290 × 120 mm, TR/TE/TI 5.1/1.5/250 ms, flip angle = 25°, acquired resolution 1.1 × 1.4 × 2.0 mm, TFE factor 19–26, parallel imaging factor 2, and spectral fat suppression. Trigger delay was matched to the beginning of the quiescent time in diastole as determined from a CINE SSFP image through the aortic root (typically left ventricular outflow tract view). Acquisition duration was chosen by the technologist to best match the duration of the quiescent diastolic period and typically varied between 95 and 130 ms. Contrast was dosed according to our institution's standard policy: gadofosveset trisodium 10 or 15 mL (15 mL for patients > 85 kg) and gadobenate dimeglumine uniformly 20 mL. Patient demographics and dosing are summarized in Table 1, with the average weight-based dose being 0.137 mmol/kg for gadobenate dimeglumine (standard dose 0.1 mmol/kg) and 0.033 mmol/kg for gadofosveset trisodium (standard dose 0.03 mmol/kg). All SS-MRA studies were initiated within 3-4 minutes of contrast injection. Nominal scan times were 4–5.5 minutes (depending on gating parameters), with navigator efficiencies typically ranging from 40 to 60% resulting in total scan times of 6.5–13 min.

**Figure 1:**
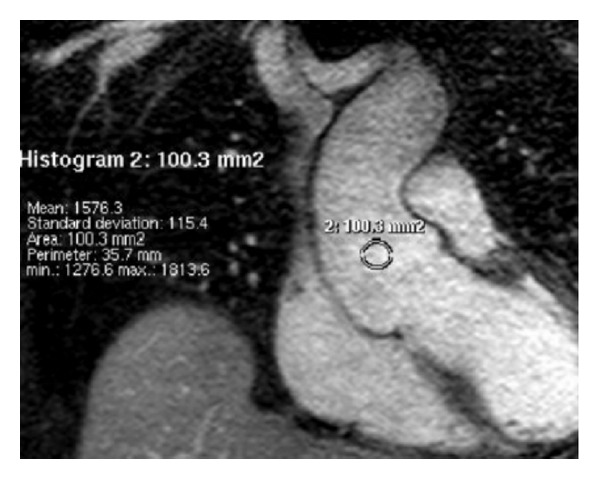
SNR was estimated as ratio of signal intensity to standard deviation, after ROI placement in a vessel of interest (in this case ascending aorta, As Ao, circular region above).

For evaluation of intravascular signal-to-noise ratio (SNR), a small region of interest (ROI) was manually placed in the most homogeneous possible regions of the following structures: right atrium (RA), left ventricle (LV), left atrium (LA), ascending aorta (As Ao), descending aorta (Desc Ao), and right pulmonary artery (RPA) (Figure 1). All ROIs were placed by a cardiothoracic imaging fellow and then reviewed by a cardiovascular radiologist with greater than 10 years of experience. Mean signal intensity (SI) and standard deviation (SD) were recorded at each location for both groups. SNR was estimated as the ratio of SI/SD.

**Figure 2:**
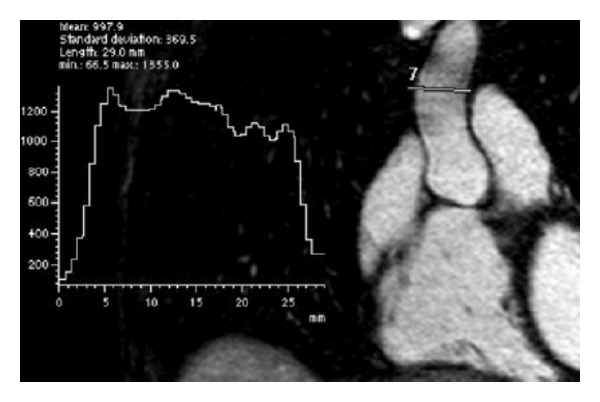
Vessel edge sharpness was determined as the distance (mm) of the 20–80% rise as derived from line intensity profile perpendicular to vessel of interest (ascending aorta, above).

Vessel edge sharpness (ES) was determined as the 20–80% rise distance (mm) derived from line intensity profiles perpendicular to the left pulmonary vein (LPV), ascending aorta (As Ao), and descending aorta (Desc Ao) (Figure 2). Full range was measured from background extravascular SI to mean SI across the vascular structure of interest. As with SNR ROIs, the line intensity profiles were generated by the same cardiothoracic imaging fellow and reviewed by the same cardiovascular radiologist. All data were analyzed using a nonpaired Student's *t*-test for the determination of statistical significance with a threshold for significance set at *P* < 0.05.

## 3. Results

**Figure 3:**
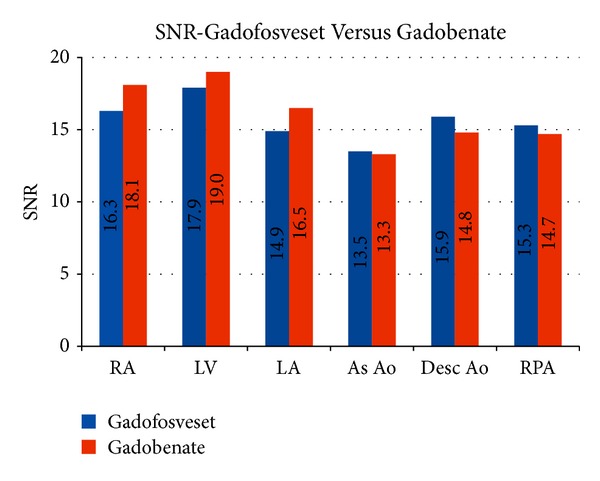
Mean SNR in different vascular structures for gadofosveset and gadobenate groups. Right atrium (RA), left ventricle (LV), left atrium (LA), ascending aorta (As Ao), descending aorta (Desc Ao), and right pulmonary artery (RPA).

All studies were of diagnostic quality. Mean SNR in the RA, LV, LA, As Ao, Desc Ao, and RPA for gadofosveset trisodium and gadobenate dimeglumine groups were 16.3 and 18.1, 17.9 and 19.0, 14.9 and 16.5, 13.5 and 13.3, 15.9 and 14.8, and 15.3 and 14.7, respectively (Table 2, Figure 3). There was no statistically significant difference in the mean SNR for any vessel between the two groups with P values ranging from 0.14 for SNR values in the RA to 0.85 for SNR values in the As Ao.

**Figure 4:**
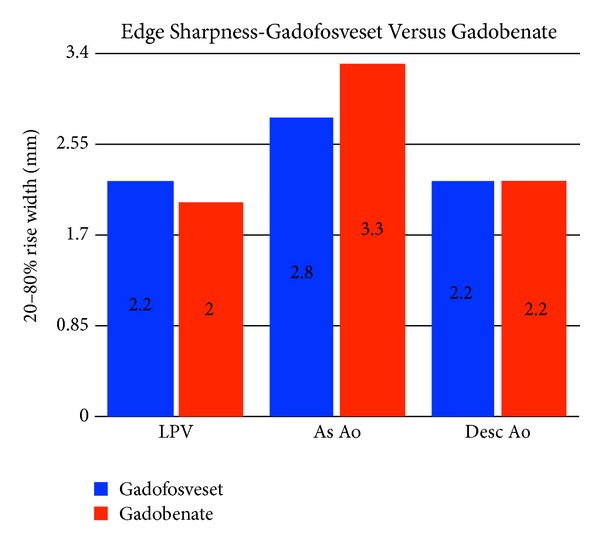
Edge sharpness for SS-MRA using gadofosveset and gadobenate measured in three vessels. Left pulmonary vein (LPV), ascending aorta (As Ao), and descending aorta (Desc Ao).

Mean vessel edge sharpness in millimeters (mm) for the LPV, As Ao, and Desc Ao for the gadofosveset trisodium and gadobenate dimeglumine groups were 2.2 and 2.0, 2.8 and 3.3, and 2.2 and 2.2, respectively (Table 3, Figure 4). There was no statistically significant difference in the mean vessel ES between the two groups, with P values ranging from 0.17 for the As Ao to 0.78 for the Desc Ao.

**Figure 5:**
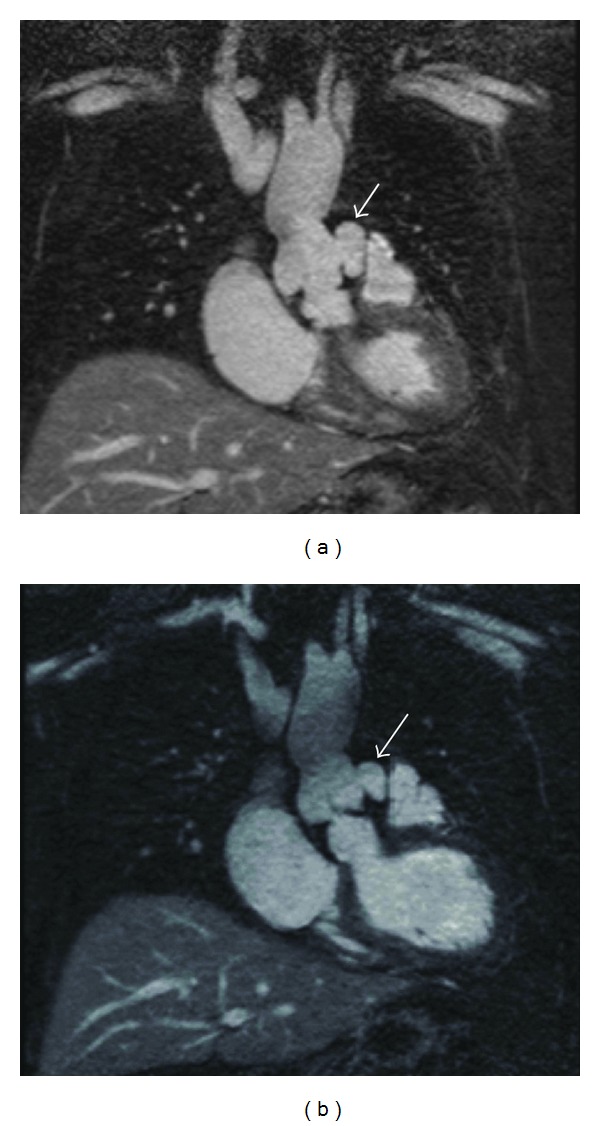
Coronal source images for a (nonstudy) patient with a left sinus of Valsalva aneurysm (arrows) performed using (a) gadobenate dimeglumine and (b) gadofosveset trisodium 6 months later (at which time slightly more thrombus is seen within the aneurysm). Note the very similar overall image quality for both contrast agents, with excellent depiction of the aortic root detail. Note also some mild shading of the ascending aorta (b) that is an occasional artifact related to fat suppression.

Figure 5 demonstrates a comparison between agents where the same patient was imaged with gadobenate dimeglumine and gadofosveset trisodium six months apart (nonstudy patient). Note the similar image quality and sharpness obtained with the different contrast agents.

## 4. Discussion

Given the increased imaging times inherent to acquiring high-resolution SS-MRA, the blood pool contrast gadofosveset trisodium has naturally become a preferred agent for steady state imaging due to its high T1 relaxivity and prolonged intravascular retention as compared to standard extracellular GBCAs [13, 14]. Thus we initially hypothesized that gadofosveset trisodium would exhibit significantly improved SNR and vessel edge sharpness for thoracic SS-MRA as compared to the extracellular agent gadobenate dimeglumine. On the contrary, we found no significant difference in our two measures of “image quality”—SNR and vessel edge sharpness. The fact that this study did not show any statistical difference in SNR or ES between the agents can likely be explained by (a) the similarity of net injected T1 relaxivity (i.e., net “efficacy” of T1 shortening) between gadofosveset trisodium and gadobenate dimeglumine and (b) the rapid initiation of SS-MRA imaging (beginning within 3-4 minutes of injection).

Blood T1 shortening can be approximated to the product of T1 relaxivity (r_1_) and blood GBCA concentration. While the T1 relaxivity of gadofosveset trisodium is approximately three times that of gadobenate dimeglumine (6.3 versus 19 L mmol^−1^ s^−1^ at 1.5T according to [13]), the injected dose of gadobenate dimeglumine is approximately four times greater in our case (0.137 versus 0.033 mmol/kg). Thus, one would expect that the early equilibrium T1 values for both agents are roughly equal or even favor gadobenate dimeglumine up to the point of significant differential extracellular extravasation. Per package inserts [14, 15], the distribution (intravascular) half-life for gadobenate dimeglumine is 5–36 min, while that for gadofosveset trisodium is 30 min. Thus even taking the lower limit 5-minute intravascular half-life for gadobenate dimeglumine, at least comparable T1 shortening several minutes into equilibrium would be expected between gadobenate dimeglumine and gadofosveset trisodium. Based on the lack of observed SNR difference between the two agents for our studies initiated within 3-4 minutes (and lasting up to 13 min), the “effective” gadobenate dimeglumine half-life appears to be well beyond 5 minutes for this application. This may be in part due to the weak protein binding properties of gadobenate dimeglumine [16].

Of important consideration, it has been our anecdotal experience that when initiating SS-MRA with gadobenate dimeglumine much beyond the time frame of 3–5 minutes (e.g., if a technical problem occurs and the SS-MRA needs to be repeated 5–10 minutes later), SNR and image quality are significantly degraded. Under these circumstances, or when it is anticipated that slow or delayed imaging will occur, or when there is a need to image a second vascular territory, gadofosveset trisodium would be the preferred agent. Further definition or exploration of this, however, was beyond the scope of this study.

Likewise, we initially anticipated that vessel edge sharpness may decrease with gadobenate dimeglumine due to potential more rapid contrast extravasation into the soft tissues of the vessel wall, where such wall enhancement may alter the slope (sharpness) of the lumen edge as measured. Although there was a slight trend toward this effect seen only in the ascending aorta (Figure 4), this does not appear to be the case given that no statistical difference was found in the three vessels we investigated.

SS-MRA of the thoracic vasculature provides improved image quality, yielding high-resolution imaging of both the arteries and veins [8]. Using ECG gating and navigator triggering to compensate for respiratory and cardiac motion, we have found SS-MRA to be a useful supplement to FP-MRA, especially for evaluation of the aortic root. In particular, we use SS-MRA to assess coronary artery origins, ascending aortic pathology such as aneurysm, fistula, and dissection, and complex vascular anatomy in our adults with repaired congenital heart disease. As is well known, conventional FP-MRA is more subject to cardiac pulsation artifacts and therefore is less accurate than ECG-triggered MRA techniques, making the latter more desirable for accurate and reproducible measurements close to the highly dynamic aortic root [17, 18].

Although this is, to our knowledge, the first study evaluating the use of an ECF agent for SS-MRA of the extra-cardiac thoracic vasculature, others have examined the use of gadobenate dimeglumine for whole heart/coronary MRA [19, 20]. In a somewhat comparable intraindividual comparison between single dose gadofosveset trisodium and gadobenate dimeglumine at 3 T, Raman et al. demonstrated no significant SNR difference between the two agents, although they noted slightly better subjective image quality for the gadofosveset trisodium [20]. Furthermore, several recent studies have similarly demonstrated the feasibility of gadobenate dimeglumine for steady state MRA outside the thorax, with Anzidei et al. establishing that high quality SS-MRA can be obtained in both the carotid and peripheral vasculature [21, 22]. In another study, Christie et al. demonstrated that nearly equal image quality can be obtained with either gadofosveset trisodium or gadobenate dimeglumine (single dose, parallel group study) when performing lower extremity SS-MRA [23]. That these studies report initiating steady state imaging immediately after first pass imaging [21], or following the slow administration of small amount of additional contrast [22, 23], corroborates our assertion that rapid initiation of imaging postcontrast administration is vital to the success of gadobenate dimeglumine SS-MRA.

The results of our study demonstrate that high quality thoracic SS-MRA does not require a blood pool agent and can be performed using the extracellular GBCA agent gadobenate dimeglumine if begun in the early equilibrium phase. As described, this likely relates to a combination of gadobenate dimeglumine's high T1 relaxivity (as compared to other ECF agents—a function of its weak and transient protein binding to serum albumin [13, 16]) and (while not being a true “blood pool” agent) its sufficient intravascular half-life to sustain steady state imaging over many minutes, which may also be an effect of its weak protein binding [7]. While not being formally explored as part of this work, it is noteworthy to report that in our experience attempts to perform thoracic SS-MRA with other conventional ECF agents (e.g., gadoteridol) yield much less satisfactory results, presumably due to their lesser T1 relaxivity. Of further clinical relevance, the use of an extracellular GBCA, such as gadobenate dimeglumine, offers the added benefit over a blood pool agent of providing parenchymal evaluation, such as delayed myocardial enhancement.

This study has several limitations. First, it is limited by virtue of its relatively small size (40 patients; 20 with each contrast agent). Second, by nature of its retrospective design, it was a parallel group study, meaning that imaging with both contrast agents was not performed on the same patient. Third, the clinical choice of contrast agent (blood pool versus ECF) was made by the radiologist who protocoled the case and was in part determined by whether other imaging such as delayed enhancement was desired, or which agent had been used on prior exams. Thus, there may be some selection bias in the type of patient who received one type of contrast versus the other. There was no significant difference in patient age or weight (Table 1). Fourth, SNR was estimated as the mean signal in the vessel of interest divided by the standard deviation within that ROI. This is not the optimal way to measure SNR given possible SI inhomogeneities distorting the standard deviation, and a more accepted way is to use the standard deviation of air as noise. Unfortunately this could not be done, as the relatively tight coronal acquisition excluded air from the images in most cases, and furthermore scans using parallel imaging are known to have highly spatially dependent noise [24]. The relative uniformity of measured SNR across the different vascular structures (Figure 3) gives some assurance that heterogeneity of intravascular signal intensity did not dramatically alter the measured SNR. Finally, and perhaps most importantly, by virtue of how contrast was prescribed according to the then existing hospital policies, the doses for each contrast agent were not weight-based and therefore perfectly “standard,” but instead “volume based,” with gadobenate dimeglumine administered as a uniform 20 mL bolus but gadofosveset trisodium given as either 10 or 15 mL depending upon patient body weight (15 mL dose if >85 kg). This is a limitation we are stuck with due to the retrospective nature of the study design. As can be seen from Table 1, the average dose of gadobenate dimeglumine was 0.137 mmol/kg, or 37% greater than its “standard” dose (0.1 mmol/kg), whereas the average dose of gadofosveset trisodium was 0.033 mmol/lg, or 10% greater than its “standard” dose (0.03 mmol/kg). Thus it could be argued that there was a slight advantage in dose for gadobenate dimeglumine, and if these studies were both performed at “standard” dose it is possible that there may be a small SNR differential favoring gadofosveset trisodium. It should be noted, however, that while both agents are FDA approved for MRA in specific vascular territories, no agent is considered “approved” for thoracic MRA, and hence there is no “approved dose” for either agent [14, 15].

## 5. Conclusion

High quality thoracic ECG-triggered SS-MRA comparable to that achieved using the blood pool agent gadofosveset trisodium can be obtained with the use of the high relaxivity extracellular agent gadobenate dimeglumine provided that a slightly higher dose (20 mL, or approximately 0.14 mmol/kg) is administered and that imaging is initiated relatively quickly (3-4 min) after contrast injection.

## Figures and Tables

**Table 1:** Tabular description of patient demographics and dosing for each contrast agent. No significant difference in patient age or weight between groups. Statistically significant dose difference (∗) is calculated based on dose relative to “standard dose” of 0.03 mmol/kg gadofosveset trisodium and 0.1 mmol/kg gadobenate dimeglumine.

	Gadofosveset	Gadobenate	*P*-value
Number of patients	20	20	N/A
Gender	15 M/5 F	9 M/11 F	N/A
Average age—years (SD)	39 (15)	44 (17)	0.40
Average weight—kg (SD)	81 (17)	79 (26)	0.75
Average dose—mmol/kg (SD)	0.033 (0.006)	0.137 (0.033)	0.003∗

**Table 2:** Mean SNR and standard deviation (SD) in different vascular structures for gadofosveset and gadobenate cohorts with *P*-values and 95% confidence intervals.

	Signal-to-noise ratio (SNR)			95% confidence interval	*P*-value
	Mean (SD)	Mean (SD)	Difference		
	Gadofosveset	Gadobenate			
RA	16.3 (3.7)	18.1 (4.0)	1.8	(−0.6, 4.3)	0.14
LV	17.9 (5.6)	19.0 (4.5)	1.0	(−2.2, 4.3)	0.52
LA	14.9 (3.3)	16.5 (4.0)	1.7	(−0.7, 4.0)	0.17
As Ao	13.5 (3.2)	13.3 (3.4)	−0.2	(−2.3, 1.9)	0.85
Desc Ao	15.9 (4.0)	14.8 (3.7)	−1.1	(−3.6, 1.3)	0.35
RPA	15.3 (3.4)	14.7 (3.3)	−0.5	(−2.7, 1.6)	0.62

Right atrium (RA), left ventricle (LV), left atrium (LA), ascending aorta (As Ao), descending aorta (Desc Ao), and right pulmonary artery (RPA). See also Figure 3.

**Table 3:** Edge sharpness and standard deviation (SD) for SS-MRA using gadofosveset trisodium and gadobenate dimeglumine measured in three vessels.

	Edge sharpness			95% confidence interval	*P*-value
	Gadofosveset	Gadobenate	Difference		
	Mean (SD)	Mean (SD)			
LPV	2.2 (0.7)	2.0 (0.5)	−0.2	(−0.6, 0.2)	0.34
As Ao	2.8 (0.6)	3.3 (1.5)	0.5	(−0.2, 1.2)	0.17
Desc Ao	2.2 (0.4)	2.2 (0.6)	0.0	(−0.4, 0.3)	0.78

Left pulmonary vein (LPV), ascending aorta (As Ao), descending aorta (Desc Ao). See also Figure 4.
